# Rabbit Does as a Model for Studying Plasma Metabolomic Adaptations Across Reproductive Stages: Insights from Parturition to Weaning

**DOI:** 10.3390/vetsci13050497

**Published:** 2026-05-20

**Authors:** Jorge Mateo-López, Alejandro Huertas-Herrera, Mónica Toro-Manríquez, Diego Páez-Rosas, Mette Skou Hedemann, Lola Llobat, Pablo Jesús Marín-García

**Affiliations:** 1 Department of Animal Production and Health and Food Science and Technology (PASAPTA), Medicine Veterinary Faculty, Universidad Cardenal Herrera-CEU, CEU Universities, 46113 Valencia, Spain; jorgemateo22@gmail.com; 2 Centro de Investigación en Ecosistemas de la Patagonia (CIEP), Camino Baguales s/n Km 4.7, Coyhaique 5951601, Chile; alejandro.huertas@ciep.cl (A.H.-H.); monica.toro@ciep.cl (M.T.-M.); 3 Galapagos Science Center, Universidad San Francisco de Quito, Isla San Cristóbal, Islas Galápagos 200150, Ecuador; 4 Fundación Conservando Galápagos, Galapagos Conservancy, Isla Santa Cruz, Islas Galápagos 200350, Ecuador; 5 Dirección del Parque Nacional Galapagos, Unidad Técnica Operativa San Cristóbal, Isla San Cristóbal, Islas Galápagos 200150, Ecuador; 6 Department of Animal and Veterinary Sciences, Aarhus University, Blichers Alle 20, DK-8830 Tjele, Denmark; 7 Molecular Mechanisms of Zoonotic Diseases (MMOPS) Research Group, Department of Animal Production and Health and Food Science and Technology (PASAPTA), Medicine Veterinary Faculty, Universidad Cardenal Herrera-CEU, CEU Universities, 46113 Valencia, Spain; maria.llobatbordes@uchceu.es

**Keywords:** rabbit model, molecular biology, reproductive physiology, lipid metabolism

## Abstract

Metabolomics is increasingly applied in veterinary molecular biology to investigate physiological adaptations in animals. In this study, rabbit does were used as a model to explore plasma metabolomic changes associated with key reproductive stages, specifically parturition and weaning. Using an untargeted metabolomics approach, 48 plasma samples were analyzed to characterize metabolic differences between these physiological states: parturition (*n* = 24) and weaning (*n* = 24). The experiment was conducted between February and November 2019. Distinct metabolomic profiles were observed between stages, with variations detected in metabolites associated with lipid metabolism, energy homeostasis, and cellular metabolic pathways. Distinct changes included higher plasma levels of Betaine and alpha-CEHC at parturition, while weaning was characterized by elevated levels of 4-Pyridoxic acid, Proline betaine, Allysine, modified phospholipids, and other nitrogenous and lipophilic metabolites, reflecting stage-specific metabolic adaptations. These results contribute to the understanding of metabolic regulation during reproduction and lactation in mammals and highlight the usefulness of the rabbit as an experimental model in veterinary molecular and physiological research.

## Simple Summary

This study examines plasma metabolomic changes in rabbit does between parturition and weaning using an untargeted LC-MS approach on 48 samples. Distinct metabolic profiles were observed between stages, with parturition characterized by higher levels of metabolites such as betaine and alpha-CEHC, while weaning showed increased levels of compounds related to amino acid, vitamin B6, and lipid metabolism, including 4-pyridoxic acid, proline betaine, allysine, and modified phospholipids. These results highlight significant metabolic adaptations associated with reproductive stage, particularly in pathways related to energy balance and nutrient metabolism, and support the rabbit as a useful model for studying physiological changes during reproduction and lactation.

## 1. Introduction

Between 70 and 80% of total production costs in rabbit farms are attributed to feed [1], which has prompted significant genetic improvement efforts aimed at increasing feed efficiency. The feed conversion ratio is one of the main traits of interest. However, selection for this trait is complex and is usually approached indirectly through selection for growth rate, due to a negative genetic correlation between the two traits. Concurrently, from a production management perspective, adjustments in feed quality and specificity play a key role in performance and the economic sustainability of the farm. In this context, it is essential to characterize these effects at each productive stage, as their impact may vary throughout the production cycle.

In this context, improving the understanding of how genetic selection influences key reproductive milestones in females remains essential. Our study focuses on two critical events in the reproductive cycle: first parturition and weaning.

Parturition represents a crucial stage in rabbit development, as various maternal factors can significantly affect fetal outcomes and neonatal viability. During the pre-partum, parturition, and postpartum stages, concentrations of key hormones, such as cortisol, glucocorticoids and progesterone, fluctuate considerably [2,3,4]. Uterine blood pressure also exhibits asynchronous variations, ranging from 0.0013 to 0.046 atmospheres [5]. Successful birth and stress responses in does are largely influenced by oxytocin levels, which activate prostaglandins as endogenous myometrial stimulants [4].

Weaning also represents a significant physiological challenge for female rabbits. Contributing factors include abrupt hormonal changes—such as a rapid decline in prolactin levels following the abrupt cessation of suckling stimulus—alterations in nutritional demands and stress in the doe. These processes can impact both physiological homeostasis and overall animal welfare [6,7].

Metabolomics has greatly enhanced our understanding of physiological processes in living organisms. This approach allows researchers to move beyond the analysis of a limited set of metabolites toward a comprehensive characterization of the entire metabolome. Such a global perspective offers a more in-depth understanding of the physiological changes occurring across different species, including rabbits [8,9,10,11,12,13,14,15].

Parturition and weaning were selected as representative physiological stages because both involve marked endocrine, metabolic, and lactational adaptations that are essential for maintaining reproductive performance and maternal homeostasis [16]. Therefore, these stages provide a relevant framework to characterize plasma metabolomic changes associated with reproductive transitions and to better understand the potential metabolic implications linked to selection for productive traits in rabbit does [17]. Rabbits represent a valuable experimental model in reproductive and metabolic research due to their high reproductive efficiency, relatively short reproductive cycle, ease of handling, and well-characterized physiology. In addition, rabbit does exhibit marked metabolic and endocrine adaptations during reproduction and lactation, making them a suitable model for investigating physiological changes associated with these stages.

Despite increasing use of genetic selection, little is known about its metabolic implications during reproductive transitions in rabbit does. This work hypothesized that rabbit does exhibit distinct plasma metabolomic profiles between parturition and weaning due to the different physiological, endocrine, and metabolic demands associated with each reproductive stage. Therefore, the objective of this study was to characterize and compare the plasma metabolomic profiles of reproductive rabbit does at parturition and weaning using an untargeted metabolomics approach, in order to identify metabolites and metabolic pathways associated with these key physiological transitions.

## 2. Materials and Methods

### 2.1. Animal Ethics Statement

All animal-related procedures received prior approval from the Animal Welfare Ethics Committee of the Universitat Politècnica de València (Approval No. 2015/VSC/PEA/00.061). The experiments were carried out in accordance with the guidelines established by the European Group on Rabbit Nutrition and complied with Directive 2010/63/EU/EEC, as well as Spanish Royal Decree 53/2013 concerning the protection of animals used in scientific research.

### 2.2. Animals and Experimental Design

The experimental units were defined as the doe rabbits together with their litters. All animals belonged to the R paternal line, which has been maintained and selectively bred at the Universitat Politècnica de València. They were kept in the university’s experimental rabbit facility under standardized environmental conditions, with homogeneous distribution throughout the building (mean daily temperatures ranging from 12.2 to 26.6 °C) and a photoperiod of 16 h light and 8 h darkness. At 63 days of age, females were individually assigned to breeding cages (700 × 500 × 320 mm). Artificial insemination was performed at 19 weeks of age, and an external nest box (220 × 350 × 370 mm) was provided from day 28 of gestation until weaning.

Before the first parturition, all females were offered the same commercial diet designed for young reproductive rabbits, supplied ad libitum (9.9 MJ digestible energy, 120 g digestible protein, and 480 g neutral detergent fiber per kg DM). On the day of the first parturition, an initial blood sample (*n* = 24) was obtained from the same 24 does that were later sampled at weaning, ensuring a repeated-measures design. Blood was collected from the central ear artery (1 mL in EDTA tubes), centrifuged at 700× *g* for 5 min, and the plasma was stored at −80 °C until further analysis. From parturition until the first weaning (28 days postpartum), females were fed a commercial diet formulated for adult reproductive rabbits ad libitum (12.3 MJ digestible energy, 148 g digestible protein, and 359 g neutral detergent fiber per kg DM). Litter size was standardized to six kits at the first parturition and to seven kits in subsequent parturitions in order to balance lactation demands, minimize variability, and improve statistical accuracy [18]. At weaning, a second blood sample (*n* = 24) was collected following the same protocol.

### 2.3. Plasma Metabolomic Analysis by LC-MS

#### 2.3.1. Solvents and Chemical Standards for Metabolomic Analysis

All plasma samples were subjected to untargeted metabolomic profiling. The solvents used throughout the procedure comprised HPLC-grade acetonitrile (VWRWest Chester, PA, USA), formic acid (Fluka, Merck KGaA, Darmstadt, Germany), and Milli-Q water. During sample preparation, internal standards—glycocholic acid (glycine-1-^13^C) and 4-chloro-DL-phenylalanine (Sigma, Merck KGaA, Darmstadt, Germany)—were introduced.

Individual processing was applied to each sample. Protein precipitation was initiated by adding 450 µL of ice-cold acetonitrile to 150 µL of plasma containing the internal standards at a final concentration of 0.01 mg/mL. The resulting mixtures were placed into 1 mL 96-well plates, vortex-mixed for 1 min, incubated at 4 °C for 10 min, and then centrifuged at 2250× *g* for 25 min at 4 °C.

After centrifugation, around 400 µL of the supernatant was carefully recovered and subjected to vacuum filtration using 96-well filter plates (Phenomenex). The filtrates were subsequently distributed into two separate 96-well plates (65 µL per well) and dried under vacuum conditions for approximately 2.5 h at 30 °C and 805× *g*. The dried residues were then reconstituted to their original volume with a H_2_O:ACN:FA (95:5:0.1, *v*/*v*/*v*) solution. The plates were sealed and centrifuged once more under the same conditions.

Chromatographic separation was carried out on a UHPLC system (Nexera X2 LC) coupled to an LCMS-9030 Q-TOF mass spectrometer (Shimadzu, Kyoto*,*, Japan), operating in both positive and negative electrospray ionization modes. An Acquity HSS T3 column (1.7 µm, 100 × 2.1 mm) was used for separation, maintained at 40 °C, while samples were stored at 10 °C. A 3 µL injection volume was applied.

The mobile phase consisted of a binary solvent system: solvent A (water with 0.1% formic acid) and solvent B (acetonitrile with 0.1% formic acid), delivered at a flow rate of 0.4 mL/min. A linear gradient was applied, increasing from 5% to 100% solvent B over 12 min, followed by a 1 min hold and a 3 min re-equilibration, resulting in a total run time of 16 min.

Mass spectrometric acquisition was performed in full-scan (MS) and data-dependent MS/MS modes across an m/z range of 50–1000. Instrument settings included an ion source temperature of 300 °C, a capillary temperature of 250 °C, and a heat block temperature of 400 °C. The electrospray voltage was set to −3.5 kV in negative ion mode. Nebulizing and drying gas flows were maintained at 3 and 10 L/min, respectively, with a detector voltage of 2.02 kV. MS/MS data acquisition was configured to select up to 10 precursor ions per cycle, using a collision energy of 20 eV (±10 eV). External calibration was performed using a 400 ppm standard solution in methanol, and data acquisition was performed using LabSolutions software (v5.114).

#### 2.3.2. Sample Quality Control and Metabolomics Data Pre-Processing

Quality control (QC) samples were generated by pooling small aliquots from all individual samples included in the study and were processed following the same protocol as the experimental samples. These pooled QC samples were periodically injected along the analytical run to evaluate instrument stability and account for possible signal drift. QC samples were injected every five samples throughout the analytical sequence to monitor instrument stability and signal drift. In addition, blank samples were analyzed to identify potential contaminants or carryover effects, while the injection sequence of all samples was randomized to minimize systematic bias.

Raw data were processed using MS-DIAL v5.5.251021 software, including peak detection, alignment, and gap filling, and metabolite annotation was performed using the software’s integrated library. The resulting dataset was exported to Excel, where features detected in blank samples were excluded. Only those signals falling within the predefined chromatographic retention time window and presenting m/z values below 700 were considered for subsequent analyses.

Data quality assessment and outlier detection were initially conducted through principal component analysis (PCA) using MetaboAnalyst. Raw LC-MS data were processed prior to statistical analysis including feature detection, alignment, and initial filtering based on detection frequency across samples and presence in quality control (QC) samples. Features with a high proportion of missing values were removed, and remaining missing values were imputed using appropriate methods implemented in MetaboAnalyst. Data were then normalized to reduce technical variation, log-transformed, and scaled prior to multivariate analysis. Signal drift and batch effects were monitored using pooled QC samples, and correction was applied when necessary based on QC-based signal correction procedures. Additionally, overall data quality and model robustness were further evaluated through permutation testing to assess the risk of overfitting in supervised models.

Subsequently, partial least squares discriminant analysis (PLS-DA) was applied to metabolites that discriminate between experimental groups. Model validation was performed through repeated random subsampling, and its performance was evaluated based on R^2^ and Q^2^ values. Relevant variables were selected according to their variable importance in projection (VIP) scores, and cross-validation was carried out using a five-fold approach with a maximum of five components. Only metabolites that met both a VIP score threshold (>1) and were statistically significant after false discovery rate (FDR) correction (*p* < 0.05) were considered for further analysis.

To further reduce the risk of model overfitting, feature selection and model evaluation were performed within a cross-validation framework to ensure independence between training and testing subsets.

#### 2.3.3. Metabolite Identification

Metabolite tentative identification was performed by matching accurate mass measurements, fragmentation spectra, and LC-MS characteristics with corresponding entries in the Human Metabolome Database (HMDB). Metabolite annotation followed the guidelines established by the Chemical Analysis Working Group of the Metabolomics Standards Initiative [19]. Level 1 identifications were validated by comparison of accurate mass (m/z), retention time, and MS/MS spectra against commercially available reference standards. In the absence of standards, metabolites were assigned as putatively annotated (level 2) when both m/z values and MS/MS fragmentation patterns matched entries in the HMDB or METLIN databases (accessed on 15 March 2025), whereas putative characterization (level 3) was applied when only m/z information was available. Features that could not be annotated were classified as level 4, reflecting low-confidence signals. For the purposes of this work, only level 2 annotations were considered for further analysis.

#### 2.3.4. Statistical Analysis of Metabolites

The statistical analysis for each identified metabolite was performed using a mixed-model approach. The physiological stage (parturition and weaning) was included as a fixed effect, and the individual animal was considered as the repeated subject to account for within-animal correlations across time points.

Models were fitted using the PROC MIXED procedure in SAS (2009), applying a repeated measures framework. The most appropriate covariance structure was selected based on the Schwarz Bayesian Criterion, following the guidelines described in 19], with Compound Symmetry identified as the best-fitting structure. Least-squares means were compared using t-tests, and statistical significance was declared at *p* < 0.05.

## 3. Results

**Figure 1:**
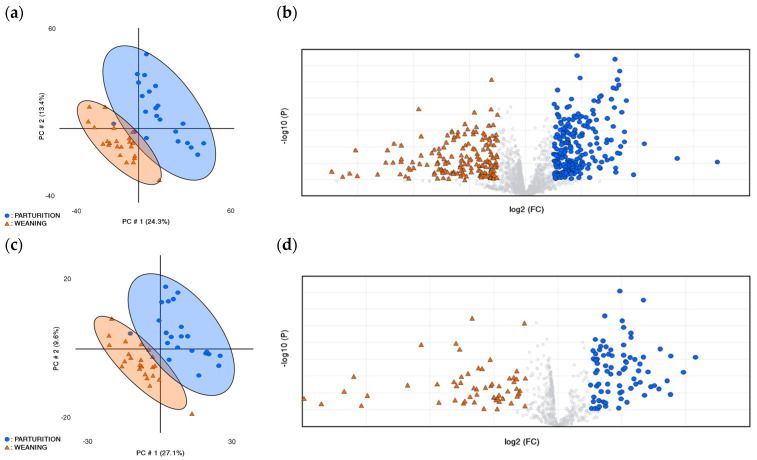
Summary of the results of the untargeted metabolomics assay regarding the effect of physiological stages in rabbit does comparing parturition and weaning time (n = 48): (**a**,**c**) Partial Least Squares Discriminant Analysis (PLS-DA) score plot of plasma in positive (ESI+, (**a**)) and negative mode (ESI-, (**c**)). The colours and shapes correspond to the two plasma extraction times: 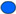: (blue) parturition time, 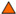: (orange) weaning time. (R^2^ = 0.80, Q^2^ = 0.65; R^2^ = 0.81, Q^2^ = 0.65, for a and c, respectively. R^2^: explained variance; Q^2^: predictive ability.) (**b**,**d**) Volcano plots show significantly different abundant metabolites between both plasma extraction times (two-sided Wilcoxon rank tests with the value adjusted by false discovery rate, FDR < 0.05 Benjamini–Hochberg) are shown; fold change threshold > 2.0 in the volcano plots. Volcano plots are in positive (ESI+, (**b**)) and in negative mode (ESI-, (**d**)).

Figure 1 illustrates the effect of physiological stage—specifically parturition and weaning across generations—on the PLS-DA score plots derived from the untargeted metabolomic analysis. The first two principal components of the PLS-DA model are presented in Figure 1a (positive mode) and 1c (negative mode), explaining 36.7% and 37.7% of the total variance, respectively. In both ionization modes, these components enable a clear separation of the experimental groups, with only minor overlap detected.

Regarding model performance, the average values obtained for goodness of fit (R^2^) and predictive ability (Q^2^) were 0.81 and 0.65, respectively. Additionally, Figure 1b,d present volcano plots highlighting the main metabolites contributing to group discrimination as identified by the PLS-DA analysis.

Table 1 summarizes the tentatively identified metabolites that explain the differences between physiological stages (parturition and weaning). At parturition, animals showed higher plasma levels of Betaine (quaternary amine derived from choline) (+28%; *p* < 0.0001) and alpha-CEHC (vitamin E degradation metabolite) (+67%; *p* < 0.0001), compared to the same animals at weaning. However, at weaning, animals showed higher plasma levels of 4-Pyridoxic acid (vitamin B6 degradation metabolite) (+290%; *p* < 0.0001), Proline betaine (quaternary amine derived from proline) (+820%; *p* < 0.0001), Allysine (metabolite derived from lysine) (+940%; *p* < 0.0001), 2-acetylpyrazine (nitrogenous heterocyclic compound) (+330%; *p* < 0.0001), LysoPE(18:3/0:0) (modified phospholipid) (+250%; *p* < 0.0001) and LysoPC (modified phospholipid) (+220%; *p* < 0.0001).

**Figure 2:**
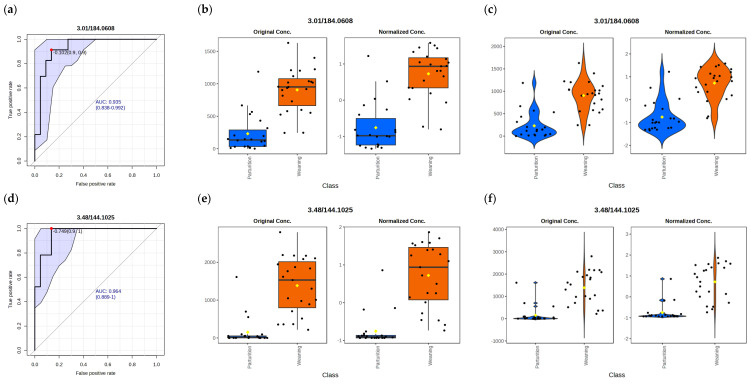

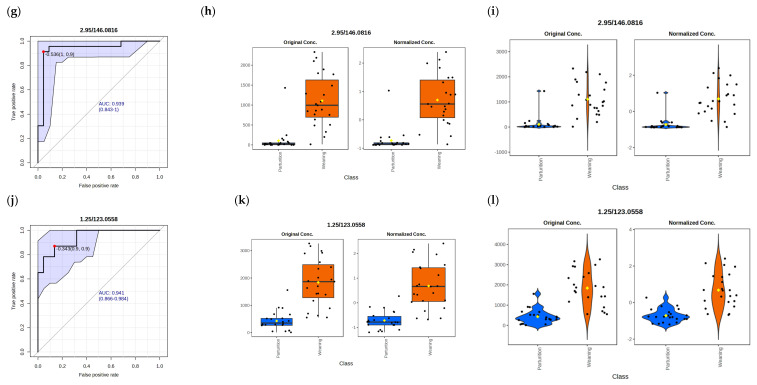

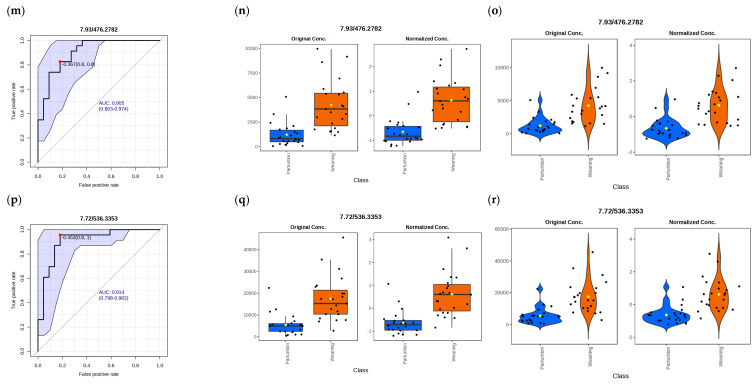

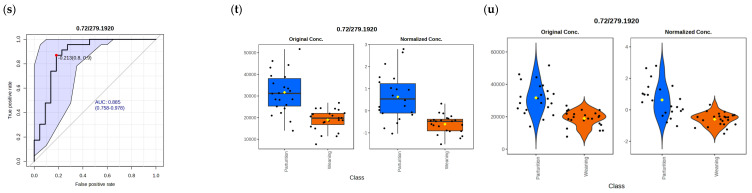
Evaluation of discriminative performance and abundance patterns of key metabolites at physiological stages (n = 48): (**a**–**c**) 4-Pyridoxic acid; (**d**–**f**) Proline betaine; (**g**–**i**) Allysine; (**j**–**l**) 2-acetylpyrazine; (**m**–**o**) LysoPE(18:3/0:0); (**p**–**r**) LysoPC; and (**s**–**u**) alpha-CEHC. For each metabolite, three complementary representations are shown in a fixed order: ROC curve (**left panels**; (**a**,**d**,**g**,**j**,**m**,**p**,**s**)), box plot (**middle panels**; (**b**,**e**,**h**,**k**,**n**,**q**,**t**)), and violin plot (**right panels**; (**c**,**f**,**i**,**l**,**o**,**r**,**u**)), illustrating both the discriminative performance between groups and the distribution of metabolite abundances. The ROC curves evaluate the ability of each metabolite to discriminate between physiological stages. Box plots and violin plots display the distribution and density of metabolite abundances across groups. The colours and shapes correspond to the two experimental groups: 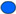: (blue) parturition time, 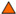: (orange) weaning time.

The discriminant ability and abundance patterns of the main metabolites are presented in Figure 2. Receiver Operating Characteristic (ROC) curves (Figure 2a,d,g,j,m,p,s), together with boxplots (Figure 2b,e,h,k,n,q,t) and violin plots (Figure 2c,f,i,l,o,r,u), are presented for those metabolites that best distinguish between the experimental groups.

The Area Under the Curve (AUC) values were consistently high, with a mean above 0.92, highlighting the strong discriminative ability of these metabolites. Proline Betaine (0.964), 2-acetylpyrazine (0.941) and Allysine (0.939) showed the highest performance.

## 4. Discussion

Our results reveal that the physiological stage significantly influenced the metabolome, as evidenced by changes in seven tentatively identified metabolites between parturition and weaning. Similar metabolic shifts between physiological stages have been consistently reported across species. In cows, Zhao et al. (2024) [20] observed that levels of non-esterified fatty acids (NEFA), β-hydroxybutyrate, and insulin increased a few days after parturition, while adiponectin decreased [20]. In contrast, Huang et al. (2023) [21] studied goats and observed that seven days after parturition, β-hydroxybutyrate, enzymes, proteins, triglycerides, and urea increased, whereas NEFA levels decreased [21]. In yaks, Shu et al. (2022) [22] reported postpartum increases in various steroids, phospholipids, and amino acid derivatives, alongside decreases in certain toxins and fatty acids [22]. In pregnant guinea pigs, Sparks et al. (1981) [23] found elevated prepartum adrenocorticotrophin, stable adrenaline, and reduced placental lactogen compared to non-pregnant controls [23]. In humans, Castro Pérez (1975) documented increased lipid metabolites during late gestation and early postpartum [24]. Similarly, Ma et al. (2024) [25] found that weaning in Bama miniature pigs led to increases in organic oxygen compounds, benzenoids, phenylpropanoids, polyketides, organosulfur compounds, and alkaloids, while organic acids, lipids, organic nitrogen compounds, organoheterocyclic compounds, and nucleosides/nucleotides decreased [25]. In rabbits, Minuti et al. (2015) [26] reported postpartum decreases in aspartate aminotransferase and increases in alkaline phosphatase [10,26], while Marín-García et al. observed elevated NEFA levels following parturition ([10]). After weaning, Bivolarski and Vachkova (2014) [27] reported reductions in cholesterol, triglycerides, triiodothyronine, and thyroxine, along with increases in creatinine and lysozyme [27]. These findings underscore significant shifts in lipid and amino acid metabolism during reproductive transitions across mammals, including rabbits.

This study aimed to elucidate the effects of reproductive stage in female rabbits. The physiological stage—parturition versus weaning—has a pronounced impact on the plasma metabolomic profile. Key affected metabolic pathways include those related to amino acid metabolism, lipid signaling, and vitamin B6 metabolism, as reflected by significant changes in metabolites such as 4-pyridoxic acid, proline betaine, allysine, 2-acetylpyrazine, LysoPE(18:3/0:0), LysoPC, and alpha-CEHC. However, rather than implying specific mechanistic roles for each metabolite, these findings should be interpreted as indicative of coordinated metabolic adjustments associated with the reproductive stage in rabbits. These findings are consistent with conserved metabolic adaptations across mammals, supporting the rabbit as a comparative model for reproductive metabolism. However, species-specific physiological and dietary differences should be considered when extrapolating these results.

Nevertheless, this study is limited by its focus on plasma metabolomics and by the potential influence of dietary changes between physiological stages. Females received one commercial diet before parturition and a different diet during lactation, so some of the metabolomic differences observed at weaning may not be related only to reproductive status but also to diet composition, lactation demands, age, or time since parturition. Accordingly, causal interpretation of the observed metabolic changes should be avoided, as the untargeted design does not allow disentangling these contributing factors. Despite this limitation, the results provide useful insight into metabolic changes during reproduction and highlight the importance of considering feeding strategy when interpreting metabolomic data in lactating does. Further targeted studies, incorporating transcriptomic and proteomic data, are warranted to clarify the underlying regulatory mechanisms and evaluate potential physiological trade-offs associated with these metabolic shifts.

## 5. Conclusions

This study sought to evaluate how the reproductive stage influences the plasma metabolomic profile of female rabbits. The main findings can be summarized as follows: (i) The shift from parturition to weaning is linked to alterations in multiple metabolites. (ii) Among the compounds tentatively identified, those related to amino acid metabolism, vitamin B6 metabolism and lysophospholipids (such as LysoPE and LysoPC) showed the most consistent differences between stages. (iii) These results suggest metabolic shifts mainly linked to lipid metabolism and energy balance, likely reflecting physiological adaptations during reproduction and lactation. Further targeted studies, including transcriptomic or proteomic approaches, are needed to better understand the underlying regulatory mechanisms and their biological significance.

## Figures and Tables

**Table 1:** List of plasma metabolites differentiating among physiological stages in rabbit does (24 animals, 48 samples).

			Parturition *		Weaning *			
RT-m/z	ION	Metabolite	X	±SE **	X	±SE	Fold ***	*p*_Value
3.01/184.0608	[M+H]^+^	4-Pyridoxic acid	230	±68	906	±66	0.254	<0.0001
3.48/144.1025	[M+H]^+^	Proline betaine	151	±127	1385	±124	0.109	<0.0001
2.95/146.0816	[M+H]^+^	Allysine	107	±110	1109	±107	0.096	<0.0001
1.25/123.0558	[M+H]^+^	2-acetylpyrazine	429	±143	1841	±140	0.233	<0.0001
7.93/476.2782	[M+H]^+^	LysoPE(18:3/0:0	1225	±426	4237	±416	0.289	<0.0001
7.72/536.3353	[M+NH_4_]^+^	LysoPC	5431	±1708	17,444	±1671	0.311	<0.0001
0.72/279.1920	[M+H]^+^	alpha-CEHC	31,693	±1598	18,947	±1563	1.673	<0.0001

(*) Parturition and weaning: two different physiological stages of the rabbit does at 0 days after birth and 28 days after birth, respectively. (**) LS means ± Standard error (***) Fold change was calculated by dividing the mean intensity of the plasma metabolite in parturition by the mean intensity of the corresponding metabolite in weaning animals.

## Data Availability

The data presented in this study are openly available. The metabolomics data have been deposited in Zenodo and are publicly accessible at DOI: 10.5281/zenodo.19297072.

## Abbreviations

The following abbreviations are used in this manuscript:

LC-MS	Liquid chromatography–mass spectrometry
PCA	Principal component analysis
PLS-DA	Partial least squares discriminant analysis
ACN	Acetonitrile
IS	Internal standards
HPLC	High-performance liquid chromatography
m/z	Mass-to-charge ratio
QC	Quality control
NMR	Nuclear magnetic resonance
GC-MS	Gas chromatography–mass spectrometry
TOF-MS	Time-of-flight mass spectrometry
FT-ICR-MS	Fourier-transform ion cyclotron resonance mass spectrometry
ROC	Receiver operating characteristic
AUC	Area under the curve
VIP	Variable importance in projection
ESI	Electrospray ionization
HMDB	Human Metabolome Database
